# Impact of COVID-19 public health restrictions on older people in Uganda: “hunger is really one of those problems brought by this COVID”

**DOI:** 10.1017/S1041610220004081

**Published:** 2020-12-17

**Authors:** Clarissa Giebel, Bwire Ivan, Philomena Burger, Isaac Ddumba

**Affiliations:** 1 Department of Primary Care & Mental Health, University of Liverpool, Liverpool, UK; 2 NIHR ARC North West Coast, Liverpool, UK; 3 African Research Centre for Ageing and Dementia, Mukono, Uganda; 4 University of Chicago, Chicago, IL, USA

**Keywords:** Ageing, COVID-19, Developing countries, public health

## Abstract

**Objectives:**

To explore the impact of COVID-19 public health restrictions on the lives of older adults living in Uganda.

**Design:**

Qualitative semi-structured interview study.

**Setting:**

Participants’ homes.

**Participants:**

Older adults living in Uganda (aged 60+).

**Measurements:**

Older adults in Uganda were interviewed over the phone and asked about their lives before and since COVID-19, and how public health restrictions have affected their lives. Semi-structured interviews were audio-recorded, transcribed and translated into English. Transcripts were thematically analyzed and themes generated in discussion.

**Results:**

In total, 30 older adults participated in the study. Five themes were identified: (1) economic impacts; (2) lack of access to basic necessities; (3) impact on healthcare utilization; (4) social impacts and (5) violent reinforcement of public health restrictions. COVID-19 public health restrictions had severe impacts on their lives, with many people having not enough food to eat due to lack of income, and being unable to pay their grandchildren’s school fees. Steep rises in public transport fares and an overall avoidance of transport also resulted in a lack of access to healthcare services and difficulty in getting food. Restrictions were violently reinforced by security guards.

**Conclusions:**

Public health restrictions have a severe impact not only on older adults but also on the whole family in Uganda. Governmental strategies to contain the virus need to provide more support to enable people to get basic necessities and live as normal a life as possible.

## Background

First reported in Wuhan, China, in December 2019, COVID-19 has spread across the globe rapidly. Amongst those most vulnerable to the virus are older adults (Shahid *et al.*, 2020), having led to shielding in many countries, in addition to public health measures of lockdown, social distancing, wearing protective masks and gloves and washing hands regularly.

Experiences of public health measures and their impacts are likely to differ between countries, possibly even more so between high- and low- and middle-income countries (LMIC). In Uganda, older adults (aged 60 and above) form 2.7 percent of the population (United Nations, 2017) and are amongst the poorest members of society without a steady income or state pension system (Kowal *et al.*, 2010). Most older people in Uganda (and generally in sub-Saharan Africa) are living with family members in multi-generational households with their children and grandchildren (Wandera *et al.*, 2017), with younger generations looking after their elderly. However, without a reliable and consistent income, and high levels of chronic conditions in older adults, accessing healthcare is very limited and often restricted to basic medicines (Nawagi *et al.*, 2018). Considering that the proportion of older adults is rising faster in LMIC, with the latest figures from 2015 of 46 million older adults in sub-Saharan Africa estimated to be more than triple by 2050 (United Nations, 2018).

LMICs are likely to be more severely impacted by COVID-19 outbreaks. This is because of poor living standards which are common and include: poor access to food, water and other basic necessities (such as medication), less well-established healthcare systems (which might be too expensive to access), as well as overcrowded living facilities (Hodkinson *et al.*, 2020). These factors can all contribute to the faster spread of the virus, and without appropriate and implemented public health measures containment might be impossible. Older adults in LMIC might particularly struggle due to poorer living conditions and potential inabilities to adhere to measures due to lack of water and sanitation facilities (Lloyd-Sherlock *et al.*, 2020). The COVID-19 pandemic is shown to have a severe impact on people’s lives, whether by directly being infected with the virus or dealing with public health restrictions (Chen *et al.*, 2020; Wright *et al.*, 2020). However, it is unclear to what extent the public health measures as such are impacting on the lives of older adults in LMIC, including Uganda.

The aim of this study was to explore the impact of COVID-19 public health restrictions (including social distancing, curfew, increased hand washing and face masks) on the lives of older adults in Uganda. To date, no research has explored the indirect effects of COVID-19 on the lives of people of any age in a LMIC. Knowledge about how COVID-19 public health restrictions are affecting some of the most vulnerable in our societies can help to identify potential strategies to supporting them better. Considering the expected longevity of COVID-19 and its impacts on people’s lives, it is important to ensure that public health measures keep people safe and ensure their well-being, and do not cause any harm.

## Methods

### Participants and recruitment

Older adults aged 60 or above living in Uganda were eligible to take part. Participants’ phone contacts were retrieved from a list of registered older persons from the Mukono district, approximately 24 km from the capital of Uganda. Participants were recruited via convenience sampling, and those who agreed to take part were interviewed in the order people had agreed to participate. Age (60 years or above) guided the sampling of the participants’ phone contacts. Ethical approval was obtained from The Aids Support Organization Research Ethic Committee [Ref: TASOREC/084/20-UG-REC-009] and individual consent approval was sought from the participants as well.

### Procedure

In-depth semi-structured interviews were conducted by two trained research assistants in June 2020. Phone call interviews lasted approximately 25–60 minutes in a free and flowing in-depth discussion and were audio-recorded. Verbal informed consent was obtained at the beginning of the interview. The interview questions to participants were asked in “Luganda”, which is a locally spoken dialect. Verbatim of all the audio-recorded interviews were transcribed in the local dialect and then those transcripts were back translated into English by the research assistants supervised by ID. This process was chosen to ensure the original meaning of the participant’s thoughts/statements, so that no response was altered or lost in the process of transcription.

### Data collection and tools

A semi-structured interview guide was developed by the team to understand the impact of public health restrictions on the well-being of older persons. The opening question focused on highlighting older adults’ living situations, how they spent their days before the COVID-19 pandemic, the impact of public health restrictions on their daily life, their perception on their inability to perform certain tasks due to the public health measures and how the public health measures have affected their social life.

### Data analysis

The analysis followed thematic analysis (Braun and Clarke, 2006) deductively focusing on how corona virus public health measures affected the well-being of older persons in Uganda. The transcripts were then entered into Atlas from where they were coded into a codebook developed by the study team. Textual data relating to the theme would be highlighted and attached to the corresponding them. A manual process of pile sorting similar quotes attached to particular codes was conducted which led to the identification of sub-themes under each code. One researcher coded by hand without software. These were summarized into mini-statements from which following a perusal process, the main themes were derived. Codes and themes were discussed amongst the team after individual coding, and final themes were agreed upon.

## Results

Thirty older adults took part in this study. Participants were mostly female (76.7%) and on average 74 years (±8, 60–88 range) old. All participants lived in multi-generational households.

Thematic analysis generated five overarching themes, with further sub-themes also highlighted in Table 1: (1) economic impacts; (2) lack of access to basic necessities; (3) impact on healthcare utilization; (4) social impacts and (5) violent reinforcement of public health restrictions. Overall, most older adults were impacted by public health restrictions in some form, whilst some people appeared to be affected very little due to lack of mobility and generally staying at home.

**Table 1.** Coding tree of thematic analysis

theme	sub-themes
Economic impacts	Lack of income
Lack of access to basic necessities	Food
	Medicines
	Education
	Transport
Impacts on healthcare utilization	
Social impacts	Inability to see family and friends
	Religious ceremonies
Violent reinforcement of public health restrictions	

### THEME 1: Economic impacts

#### Lack of income

Older adults often relied on their children to give them money or food, many of whom are now unable to work due to the public health restrictions. Many older adults had some farm land they maintained and worked on every day. However, due to COVID-19 public health restrictions and lack of transport, they were now unable to reach the farm and create extra produce to sell. Similarly, some participants were digging every day in their garden and usually sold some limited surplus either at a stall in front of their homes or at the nearby market. Due to transport limitations, they were unable to reach the market to sell their produce.

‘Due to the Corona Virus Disease outbreak, we are no longer getting money because the person who takes care of us used to at least go to town, bring some food and also give us some 2000 Ushs for home use to maybe buy myself some milk but I am no longer able to afford it.’ **P23 (female, 73 years)**

‘It’s entirely due to corona because I used to go to some work and earn, I used to sell some potatoes from my garden, sell it and earn money, now this is not possible but now this pandemic has stopped us from this.’ **P14 (female, 68 years)**

‘we no longer do anything we used to sell our cassava here and get some little money for survival but now we can no longer do that, we can’t even afford a piece of soap because we don’t have any money with us, the money we had saved, we have consumed all of it during this season, all this is due to the corona virus pandemic’ **P15 (female, 70 years)**

Usual buyers of their produce also did no longer show up as they themselves had no income or were unable to reach the stall.

‘Before covid I had my livestock so I would sell and pay fees but now we eat them ourselves at home because those who used to buy them are not working anymore.’ **P12 (female, 78 years)**

‘I used to work for myself, I could eat and drink, because I used to make roasted g-nuts and I sell them in this area. But when corona came, there is no moving. People have no money.’ **P24 (female, 82 years)**

Some participants also had people renting a room in their home and were suffering a lack of income from renters no longer paying their rent. Renters apparently stated that the government put a temporary hold on paying rent, which participants questioned, and had no income source.

‘Another problem is that the tenants no longer want to pay rent, they have this excuse that the government requested landlords to relax and so I have nothing to do but wait.’ **P12 (female, 78 years)**

### THEME 2: Lack of access to basic necessities

#### Food

Older adults experienced severe difficulties getting access to food during the pandemic. Many older adults were used to farming and growing their own food but were restricted due to public health measures. They were also unable to buy food at the market because of a lack of income. Whilst the government was handing out “posho” to people in need, some participants did not receive the governmental food support or it was delayed, whilst others could not eat the meal as it made them feel unwell. As a result, many people had to cut down their meals, some down to one meal a day and many were constantly hungry.

‘Maybe food is really hard to get since there is no transport. So hunger is really one of those problems brought by this covid.’ **P06 (female, 66 years)**

‘The price for food is now high so as a result we can no longer afford two meals a day so we end up eating one’. **P12 (female, 78 years)**

‘Even the food that was distributed by the government for us we didn’t even get, so how are we surviving given the fact that we no longer even go to the garden to get food.’ **P14 (female, 68 years)**

The curfew at 7 pm each night also caused particular difficulties, as certain food ingredients are only sold in the evening, making it impossible for people to buy certain basic food items.

‘But for now, I can’t even afford sugar because the curfew could not allow us because the maize and tea only could be sold in the evening but now, by 7 pm you have to be indoors.’ **P05 (female, 68 years)**

#### School fees

Many participants stated they were unable to pay their grandchildren’s school fees any longer, due to a lack of income. In some cases, participants had to decide on what to use their very limited funds for, if they indeed had any funds. In those instances, money was spent on food, and not on buying a newspaper for example to help the grandchildren learn how to read.

‘Corona has even affected our children with regard to their education. Our kids don’t go to school anymore and what they teach on TV they don’t understand and can’t ask questions like they do in class rooms. So it’s not helping. They just sit and watch. The other option is buying newspapers yet I can’t afford them, because if I have the money, I rather buy food other than just papers so we should pray that this pandemic ends. This has affected us a lot.’ **P08 (female, 62 years)**

‘it has really affected us because we get school fees from our gardens after selling produce, so it’s going to be very difficult for us to acquire school fees as compared to how it has been.’ **P03 (female, 60 years)**

#### Transport

Nearly every participant raised transport difficulties affecting their daily lives since the pandemic breakout, including those participants who no longer leave the house due to frailty and old age. One of the biggest difficulties was that their children were struggling to visit them and bring them food or money. For those who were reliant on public transport, all participants raised the issue of increased costs of transport since the pandemic, which has made transport unaffordable.

‘Even the transport around, I can’t send my wife to go and visit some of her relatives to give us some money like Shs.10,000 because the transport is so expensive, where we used to travel at Shs.2000,they want Shs.10,000,so this is really a problem.’ **P20 (male, 80 years)**

‘personally I had a farm which is very far from me, it’s like 4 km from here. Now my cows might even die because I don’t go there. Transport affects me a lot because I can’t go and see them. So it’s now a period of 3 months.’ **P07 (male, 72 years)**

‘if you have enough food, we share depending on how close you are with them. You can be there and someone surprises you with sugar or rice and be happy. So even if you have a relative who can give you food, you have no way of getting the food so that’s a challenge too.’ **P05 (female, 68 years)**

### THEME 3: Impact on healthcare utilization

Access to healthcare was severely diminished by the inability to board public transport and a lack of income to buy vital medicines and see healthcare practitioners. As a result, many participants have received no healthcare or medicines at all since the pandemic and are struggling with their health conditions.

‘I didn’t access the medicines due to transport issues because I could not access the hospital. It’s at the hospital where they could determine the type of medicine I need in case there are any change but now that public transport has been allowed I got some help because I accessed the hospital and I now feel better.’ **P03 (female, 60 years)**

‘I don’t have access to medical care anymore because I can’t move to go to the hospital due to the problem of transport. It’s really hard and if am to get from the nearby clinics, the medicines are also very expensive. So when I get help from people, I get some pain killers and I survive on that.’ **P06 (female, 66 years)**

‘We can’t go to the hospitals, so you remain home and feel the pain. You buy some tablets, swallow and then sleep. We don’t have transport means, and you can’t accept to be carried on the back. So, you stay home and send for tablets, or the doctor tells you to buy some tablets.’ **P25 (female, 66 years)**

One thing that also emerged was that participants predominantly saw treatments in the form of medicine, and no other form of non-invasive treatments, such as physiotherapy, occupational therapy, changes in lifestyle or other ways to help alleviate some of their health concerns.

‘maybe treatment, getting medicines is really hard because some of us used to get our medicines from [name] hospital, [name] hospital but now we have no means to get there because public transport was closed. So that’s a very big challenge to us.’ **P07 (male, 72 years)**

### THEME 4: Social impacts

#### Inability to see family and friends

Participants were unable to see their family and friends, due to social distancing measures and very costly public transport. Whilst they stayed in contact via phone, it did not provide the same support as seeing loved ones face-to-face. In many cases, their children are supporting them financially and/or bringing them food, which has become difficult to impossible due to the public health measures. Social distancing measures and transport issues also caused some participants to feel cut off from what is going on in their community, making people feel disconnected.

‘it has also really cut us off from accessing information about what’s happening within the community. So currently you can’t even know what’s happening within society so this is not good.’ **P15 (female, 70 years)**

#### Impact on religious practices

Participants were affected by no longer being able to go to church and pray together within their community. Whilst participants were continuing to pray at home, for those who were religious, they complained about this not being the same as they missed the social aspect of gathering together every week. Being unable to attend funerals, particularly in the time of COVID-19 and increased mortality rates, was also distressing to many participants.

‘Another effect of this lockdown due to the virus is that we cannot burry our relatives anymore. This is indeed so painful because this is culturally not correct.’ **P19 (female, 80 years)**

### THEME 5: Violent reinforcements of public health restrictions

Several older adults reported violent reinforcement of public health measures by guards that have subjected them to beatings. One of the main reasons why guards were violent was because people were out after the 7 pm curfew imposed by the government. However, with many older adults struggling to get any food during the pandemic or needing urgent medicines, they sometimes were looking for food or money after the curfew, thereby following a basic need.

‘The curfew has also affected me because sometimes you can be walking around to may be buy something and then the security guys find you and beat you up.’ **P11 (male, 65 years)**

‘since it began, I have never gone to the hospital. I get my aid from here, because I am afraid. We hear that people are being beaten.’ **P25 (female, 66 years)**

‘Even the curfew time and transport have stopped us from moving to help us get food. Imagine if the LDU’s find you moving past 7 pm, they never have mercy whether old or young, they just beat.’ **P14 (female, 68 years)**

Others reported that the curfew was beneficial, as fewer younger people were out in the evening and not getting drunk anymore, with reduced crime rates, thereby creating a more peaceful environment.

The curfew has helped us on reducing idlers on the streets. People rush home always and that’s good for all of us including the young ones. **P08 (female, 62 years)**

The crime rate has tremendously reduced in our area because it’s now hard to find someone walking past 7 pm. So the number of thieves has actually reduced during this period. **P11 (male, 65 years)**

## Discussion

This is the first study showing the impacts of COVID-19 public health restrictions on older adults in an LMIC, specifically in Uganda. Public health restrictions have had a severe impact on the lives of older adults, in many cases affecting their basic existence and making it very difficult to obtain any basic necessities.

Many older adults in Uganda and generally in LMIC belong to the poorest members of society (Golaz *et al.*, 2017). COVID-19 public health restrictions, such as social distancing, curfews and movement restrictions have caused older adults to lose the little income they had by being unable to use their farmlands and create produce to sell (where this was the case), and having no customers who would have the money to buy their produce or who are able to visit their stalls. As a result, many older adults are struggling to survive, with some having had to reduce their meals from two a day to one. As suggested by Lloyd-Sherlock *et al.* (2020), older adults in LMIC are recommended to be involved in social distancing decisions, as they may already face difficulties in obtaining food and other basic necessities. Findings from this study provide the first evidence that older adults do struggle getting basic necessities as a result of social distancing and other public health measures, such as the 7 pm curfew implemented across Uganda. Whilst it is unknown how public health restrictions are affecting the lives of older adults in other LMICs, it is important for governments to work closely with the older members of societies to ensure their basic needs are not compromised by COVID-19 measures that are meant to safeguard society, and not cause harm.

Considering the frequent multi-generational households (UNPD, 2005), these impacts are not only felt by older adults themselves but also going down to their grandchildren. The lack of income and little money available, if any, make it impossible for many to pay for their grandchildren’s school fees, with older people having to make the decision between survival (buying food) and education. Access to education is usually the responsibility of parents, but due to the multigenerational households, and us interviewing older adults, not having sufficient funds within the household equally affects older members of the family. It was a prominent topic amongst participants and was therefore important to include, as it was pertinent to them. Whilst the government apparently provides some form of food supplies, these were considered too limited for multi-generational households or often were not even received. Accessing primary, secondary and higher education in sub-Saharan Africa is unevenly distributed by household income, with those from wealthier backgrounds more likely to access secondary or higher education (Lewin, 2009; Lewin and Sabates, 2012). This will have long-term implications for society, with the youngest generation being unable to receive adequate education. Since education can be a way out of poverty for the younger generation, the current inaccessibility of it and uncertainty in regard to how long various public health restrictions are going to stay in place means children may lack vital opportunities. This will in turn prevent them from providing their elders with the necessary support in the future (Golaz *et al.*, 2017). One way to overcome this issue would be to provide free education, with the government paying school fees or generally opening up school without any costs involved.

Pre-pandemic difficulties of accessing suitable healthcare, as is the case in many LMICs (Hsiao *et al.*, 2019), have been further exacerbated by COVID-19. Many older adults needed medication or healthcare assessments, but due to the lack of income from selling their produce and increased public transport fares, amongst others, were unable to access the healthcare and treatments they needed. This lack of access can magnify existing health conditions and worsen the physical and mental health of older adults, which has also initially been reported in older Filipinos (Buenaventura *et al.*, 2020) and is a concern for many LMIC (Vahia and Shah, 2020). Therefore, governments need to work closely with older adults in their societies and generate public health measures that can better support them, without affecting their physical and mental health.

As part of this, being unable to continue social practices has had a detrimental impact on people’s lives. The cancellation of religious ceremonies has been of particular concern to participants. Faith is an important aspect of the daily routine to older Ugandans and being unable to attend weekly mass and burials of loved ones has affected them negatively. In the light of COVID-19 and increased mortality in older adults (Zhou *et al.*, 2020), being unable to attend funerals to farewell close friends and family members has particularly impacted people’s quality of life and well-being. Whilst social distancing measures need to be adhered to in order to contain the spread of the virus, religious gatherings could instead be held outside (where contagion is reduced compared to inside) with the necessary distance between churchgoers. This is corroborated by recent research from the UK showing how important face-to-face contact is to well-being and how sudden withdrawal during the COVID-19 lockdown has had negative impacts on people with dementia’s and carers’ well-being (Giebel *et al.*, 2020). With lockdowns having been lifted in most countries across the globe after the first wave, and likely further waves of lockdown and intense public health restrictions, it is important to create ways to support the mental and physical well-being of older adults.

Whilst this study benefits from a substantial sample size of older adults across Uganda, there are some limitations to consider. Older adults were interviewed from a relatively rural region of Uganda outside the capital, so that problems faced by participants might not be reflective of urban residing older people. However, we interviewed a substantial number of participants, thereby generating a representative account of problems faced by older Ugandans, many of whom reside in more rural areas throughout the country. One limitation though is that over three quarters of participants were female, therefore limiting the generalizability based on gender. Given the fact that life expectancy is higher in women than men in Uganda, according to the World Health Organization’s Global Health Observatory (2020), having a greater representation of women is likely though to be more representative of the older adult population. A further limitation is that the topic guide focused predominantly on social-economic impacts of COVID-19, as opposed to psychological impacts. Therefore, limited conclusions can be drawn on the wider psychological impacts of public health restrictions. This is furthered by a lack of information about the level of sickness of participants prior to COVID-19. To avoid possible linguistic limitations, interviews were conducted by a researcher fluent in both English and the local language.

## Conclusions

COVID-19 public health restrictions have a severe impact on the lives of older adults in Uganda, affecting their basic existence and causing the inability for them to have access to sufficient food, healthcare and education for their grandchildren. Future research needs to explore the impacts of public health restrictions on the elderly in other LMICs. These first findings already strongly support the need for governments to consider the impact of the first wave of restrictions on the lives of older adults. Future restrictions need to be created that enable older adults to have access to basic necessities. These restrictions should not cause harm but should protect the most vulnerable of our societies. However, these first findings already strongly support the notion of governments taking into account the impact of the first wave of restrictions on the lives of older adults and adjust future restrictions in future waves to enable older adults to not be short of basic necessities and for restrictions not to cause harm, but merely to protect the most vulnerable of our societies.

## Conflicts of interest

The authors declare no conflicts of interest.

## Source of funding

This study was funded by the University of Liverpool and an ODA Seed Fund awarded to CG and ID. This is also independent research funded by the National Institute for Health Research Applied Research Collaboration North West Coast (ARC NWC). The views expressed in this publication are those of the author(s) and not necessarily those of the National Institute for Health Research or the Department of Health and Social Care.

## Description of authors’ roles

CG, ID, and BW designed the interview topic guide. BW conducted the interviews. CG, BW, and PB analysed the transcripts and discussed and generated themes jointly. CG wrote the manuscript with ID drafting the Methods section. All co-authors read through versions of the manuscript and approved the final version.
